# The Eighth ECS Workshop on “Calcium Signaling in Aging and Neurodegenerative Diseases”

**DOI:** 10.3390/ijms20246263

**Published:** 2019-12-12

**Authors:** Jan B. Parys, Cláudia F. Pereira, Carlos Villalobos

**Affiliations:** 1 KU Leuven, Laboratory for Molecular and Cellular Signaling, Department of Cellular and Molecular Medicine, Campus Gasthuisberg O/N-1 B-802, Herestraat 49, 3000 Leuven, Belgium; 2 Center for Neuroscience and Cell Biology (CNC), University of Coimbra, Rua Larga, Faculty of Medicine, Polo I, 1st Floor, 3004-504 Coimbra, Portugal; cpereira@fmed.uc.pt; 3 CIBB-Center for Innovative Biomedicine and Biotechnology, University of Coimbra, Rua Larga, Faculty of Medicine, Polo I, 1st Floor, 3004-504 Coimbra, Portugal; 4 Faculty of Medicine, Azinhaga de Santa Comba, Celas, 3000-548 Coimbra, Portugal; 5 Instituto de Biología y Genética Molecular (IBGM), Universidad de Valladolid y Consejo Superior de Investigaciones Científicas (CSIC), 47003 Valladolid, Spain

**Keywords:** Ca^2+^ signaling, endoplasmic reticulum Ca^2+^, mitochondrial Ca^2+^, aging, neurodegeneration, Alzheimer’s disease, Parkinson’s disease

The European Calcium Society (ECS) is very glad to present the realization of a special issue of the International Journal of Molecular Sciences (IJMS) related to the eighth ECS workshop organized this year around the theme of “Calcium Signaling in Aging and Neurodegenerative Diseases”.

This workshop took place on September 18–20, 2019 in Coimbra (Portugal) and was organized by Cláudia Pereira, Cristina Rego, Luísa Cortes, and João Malva. The scientific committee consisted of Amanda Sierra, Carlos Duarte, Carlos Villalobos, Catarina Resende de Oliveira, Jan B. Parys, and Rodrigo Cunha.

The ECS has existed to promote research into the biology of cellular calcium since 1997 and presently has a membership of 250 researchers at all career stages, from over 30 countries, both within and outside Europe. Full information about the ECS and its activities can be found via www.calciumsociety.eu. The ECS is especially well-known for its biennial international meetings (next meeting, the 16th in the series of International Meetings: August 23–27, 2020 in Cork, Ireland), with workshops focusing on a single topic occurring in the intervening years [1]. These international meetings and workshops have taken place in various countries (Belgium, France, Germany, Italy, Poland, Slovakia, Spain, Switzerland, and the UK), and we are really glad to now include Portugal as one of the countries home to an ECS event.

Moreover, the topic of the workshop in Coimbra was particularly well chosen as ageing, and the related occurrence of neurodegenerative diseases, constitute an increasingly important problem in our society. The 2018 Ageing Report of the European Community [2] predicts an increase in the old-age dependency ratio (i.e., people aged 65 and above relative to those aged 15 to 64) in the EU from 30% in 2016 to 51% in 2070. This increase in aged people is a worldwide phenomenon. In fact, the World Health Association (WHO) expects that the proportion of the population who are 60 years or older will increase from 12% (in 2015) to 22% by 2050 [3]. Moreover, the WHO also predicts that the number of people aged over 80 years will increase from 125 to 434 million in that same time frame. Such an increase in lifespan is associated with an increased likelihood of general health problems. Neurodegenerative disorders, including Alzheimer’s and Parkinson’s diseases, arise supra-linearly with age, and represent a significant burden in terms of healthcare provision [4]. These debilitating diseases are even more dramatic in view of their seemingly irreversible progression and the lack of therapeutic tools that can ameliorate disease symptoms, let alone trigger regression. A recent review highlighted the lack of disease-modifying treatments that are currently available for Alzheimer’s disease and the failure of phase 3 trials for at least 25 compounds [5]. One of the complicating factors in the development of an efficient therapeutic approach is the fact that multiple cellular dysfunctions and co-morbidities contribute to neurodegeneration. Intracellular alterations are typically observed at the level of proteostasis/autophagic response, mitochondrial behavior, reactive oxygen species production and redox status, cell survival, as well as of Ca^2+^ homeostasis. Moreover, the fact that these various processes are widely interconnected and influence each other does not make it any easier to unravel the underlying mechanism(s).

However, it is clear that most if not all cellular functions are dependent on, or at least regulated by the complex spatio-temporal Ca^2+^ signals occurring in the cell. Examples of such Ca^2+^-regulated processes include fertilization, cell proliferation and differentiation, metabolism, muscle contraction, vesicle secretion, gene transcription, synaptic plasticity and memory formation, autophagy, and cell death. It is therefore increasingly more appreciated that the dysregulation of Ca^2+^ signals leads to important malfunctions at the cellular level, and to a plethora of pathological outcomes at the organismal level [6,7].

Interestingly, several of the above-mentioned processes regulated by Ca^2+^ are directly or indirectly important for correct brain function. An important role for intracellular Ca^2+^ in aging and neurodegenerative diseases is therefore emerging (see, e.g., recent reviews [8,9,10,11,12,13]), but a deeper understanding of the exact role of Ca^2+^ signaling in the physiological aging process, and in the occurrence and progression of various age-related pathologies is now urgently needed.

Alzheimer’s disease, Parkinson’s disease, Huntington’s disease, amyotrophic lateral sclerosis, spinocerebellar ataxia, Niemann-Pick disease, and Batten disease have already been linked to defects in Ca^2+^ handling, but neither a clear mechanistic framework for the action of Ca^2+^ in a given disease nor a unifying theory about the role of Ca^2+^ in multiple diseases have been obtained. Moreover, in view of the current lack of efficient treatments for those diseases, it is clear that new approaches have to be devised. In that sense, the possibility to modify intracellular Ca^2+^ signaling within neuronal and glial cells, and of stem cells for therapeutic purposes, is very appealing, but obviously remains to be further investigated and developed.

To obtain a better insight into the role of Ca^2+^ in aging, and in the occurrence and development of neurodegenerative diseases, 61 participants from 13 countries met in Coimbra to present and discuss their current research. In this workshop, besides the plenary lecturer Giles Hardingham (UK), 13 speakers were invited, and a further 10 participants were selected to give oral communications while 19 posters were also presented. Interestingly, IJMS provided for the workshop a “Best poster award” (winner: Riccardo Filadi) and a “Best oral communication award” (winner: Tito Calì). Furthermore, it must be mentioned that a hands-on imaging workshop delivering practical advice on techniques for Ca^2+^ measurement in neuronal cells was organized by the University of Coimbra. Finally, all attendees had the opportunity to visit the ancient campus of the University of Coimbra (founded in 1290) with its famous library and science museum, declared a world heritage site by UNESCO.

Various aspects of Ca^2+^ in aging and neurodegeneration were presented at the workshop. Further, whilst a number of different neurodegenerative diseases were discussed, there were some recurrent themes and clear take-home messages in the many presentations, including: the relationship between neuronal and glial cells and the Ca^2+^ signals arising in both cell types, Ca^2+^ handling at synapses, the regulation of Ca^2+^ sequestration and release by the endoplasmic reticulum and the role of presenilins therein, the link between Ca^2+^ fluxes from the endoplasmic reticulum to mitochondria and mitochondrial Ca^2+^ overload and neurodegeneration, the usefulness of various types of animal models in the study of neurodegeneration, the development of novel tools to measure Ca^2+^ at the subcellular level and to analyse the resulting Ca^2+^ signals, the role of Ca^2+^ in the regulation of cerebral blood flow, and the relation between Ca^2+^, lifespan, and aging.

Therefore, we are very proud to present in this special issue, not only a full meeting report [14], but also a nice slate of up-to-date reviews and research papers reflecting the groundbreaking work performed by the participants of the eighth ECS workshop on “Calcium Signaling in Aging and Neurodegenerative Diseases”.

## Abbreviations

ECS	European Calcium Society
IJMS	International Journal of Molecular Sciences
WHO	World Health Organization

## References

[1] Parys J.B., Guse A.H. (2019). Full focus on calcium. Sci. Signal..

[2] (2018). The 2018 Ageing Report: Economic & Budgetary Projections for the 28 EU Member States (2016–2070). Institutional Paper 079. https://ec.europa.eu/info/sites/info/files/economy-finance/ip079_en.pdf.

[3] (2018). World Health Organization: Ageing and Health. https://www.who.int/news-room/fact-sheets/detail/ageing-and-health#targetText=People%20worldwide%20are%20living%20longer,aged%2080%20years%20or%20older.

[4] Hou Y., Dan X., Babbar M., Wei Y., Hasselbalch S.G., Croteau D.L., Bohr V.A. (2019). Ageing as a risk factor for neurodegenerative disease. Nat. Rev. Neurol..

[5] Long J.M., Holtzman D.M. (2019). Alzheimer disease: An update on pathobiology and treatment strategies. Cell.

[6] Berridge M.J. (2016). The inositol trisphosphate/calcium signaling pathway in health and disease. Physiol. Rev..

[7] Parys J.B., Bultynck G. (2018). Calcium signaling in health, disease and therapy. Biochim. Biophys. Acta Mol. Cell Res..

[8] Pchitskaya E., Popugaeva E., Bezprozvanny I. (2018). Calcium signaling and molecular mechanisms underlying neurodegenerative diseases. Cell Calcium.

[9] Mustaly-Kalimi S., Littlefield A.M., Stutzmann G.E. (2018). Calcium signaling deficits in glia and autophagic pathways contributing to neurodegenerative disease. Antioxid. Redox Signal..

[10] Glaser T., Arnaud Sampaio V.F., Lameu C., Ulrich H. (2018). Calcium signalling: A common target in neurological disorders and neurogenesis. Semin. Cell Dev. Biol..

[11] Wegierski T., Kuznicki J. (2018). Neuronal calcium signaling via store-operated channels in health and disease. Cell Calcium.

[12] Hisatsune C., Hamada K., Mikoshiba K. (2018). Ca^2+^ signaling and spinocerebellar ataxia. Biochim. Biophys. Acta Mol. Cell Res..

[13] Chandran R., Kumar M., Kesavan L., Jacob R.S., Gunasekaran S., Lakshmi S., Sadasivan C., Omkumar R.V. (2019). Cellular calcium signaling in the aging brain. J. Chem. Neuroanat..

[14] Cortes L., Malva J., Rego A.C., Pereira C.F. (2020). Calcium signaling in aging and neurodegenerative diseases 2019. Int. J. Mol. Sci..

